# Factors associated with diabetic foot ulcers and lower limb amputations in type 1 and type 2 diabetes supported by real‐world data from the German/Austrian DPV registry

**DOI:** 10.1111/1753-0407.13531

**Published:** 2024-02-25

**Authors:** Alexander J. Eckert, Stefan Zimny, Marcus Altmeier, Ana Dugic, Anton Gillessen, Latife Bozkurt, Gabriele Götz, Wolfram Karges, Frank J. Wosch, Stephan Kress, Reinhard W. Holl, for the DPV‐Wiss‐Initiative

**Affiliations:** ^1^ Institute of Epidemiology and Medical Biometry ZIBMT, University of Ulm Ulm Germany; ^2^ German Center for Diabetes Research (DZD) Munich Germany; ^3^ Department of General Internal Medicine, Endocrinology and Diabetology Helios Clinic Schwerin Schwerin Germany; ^4^ Klinik für Diabetologie, Klinikum Dortmund Dortmund Deutschland; ^5^ Medical Clinic I, Klinikum Bayreuth Friedrich‐Alexander‐University Erlangen‐Nürnberg Bayreuth Germany; ^6^ Department of Internal Medicine Herz‐Jesu‐Hospital Muenster Germany; ^7^ Department of Internal Medicine III and Karl Landsteiner Institute for Metabolic Disorders and Nephrology Clinic Hietzing, Vienna Health Care Group Vienna Austria; ^8^ Department of Internal Medicine, Diabetes, Gastroenterology, Tumor Medicine, and Palliative Care Academic Teaching Hospital Nürtingen Tübingen Germany; ^9^ Clinic for Gastroenterology, Metabolic Disorders and Internal Intensive Medicine (Medical Clinic III), Department of Endocrinology and Diabetology University Hospital Aachen Aachen Germany; ^10^ Diabetespraxis Wosch Hanau Germany; ^11^ Diabetes, Sport and Physical Activity Working Group of the DDG Unna Germany; ^12^ Department of Internal Medicine I Vinzentius Hospital Landau Landau Germany

**Keywords:** alcohol, body mass index, hypertension, lipids, sex differences, smoking, triglycerides

## Abstract

**Aims:**

Diabetic foot ulcer (DFU) is a leading cause of lower limb amputations in people with diabetes. This study was aimed to retrospectively analyze factors affecting DFU using real‐world data from a large, prospective central‐European diabetes registry (DPV [Diabetes‐Patienten‐Verlaufsdokumentation]).

**Materials and Methods:**

We matched adults with type 1 (T1D) or type 2 diabetes (T2D) and DFU to controls without DFU by diabetes type, age, sex, diabetes duration, and treatment year to compare possible risk factors. Cox regression was used to calculate hazard ratios for amputation among those with DFU.

**Results:**

In our cohort (*N* = 63 464), male sex, taller height, and diabetes complications such as neuropathy, peripheral artery disease, nephropathy, and retinopathy were associated with DFU (all *p* < .001). Glycated hemoglobin (HbA1c) was related to DFU only in T1D (mean with 95% confidence interval [CI]: 7.8 [6.9–9.0] % vs 7.5 [6.8–8.5] %, *p* < .001). High triglycerides and worse low‐density lipoprotein/high‐density lipoprotein ratio were also associated with DFU in T1D, whereas smoking (14.7% vs 13.1%) and alcohol abuse (6.4% vs 3.8%, both *p* < .001) were associated with DFU in T2D. Male sex, higher Wagner grades, and high HbA1c in both diabetes types and insulin use in T2D were associated with increased hazard ratios for amputations.

**Conclusions:**

Sex, body height, and diabetes complications were associated DFU risk in adults with T1D and T2D. Improvement in glycemic control and lipid levels in T1D and reduction of smoking and drinking in T2D may be appropriate interventions to reduce the risk for DFU or amputations.

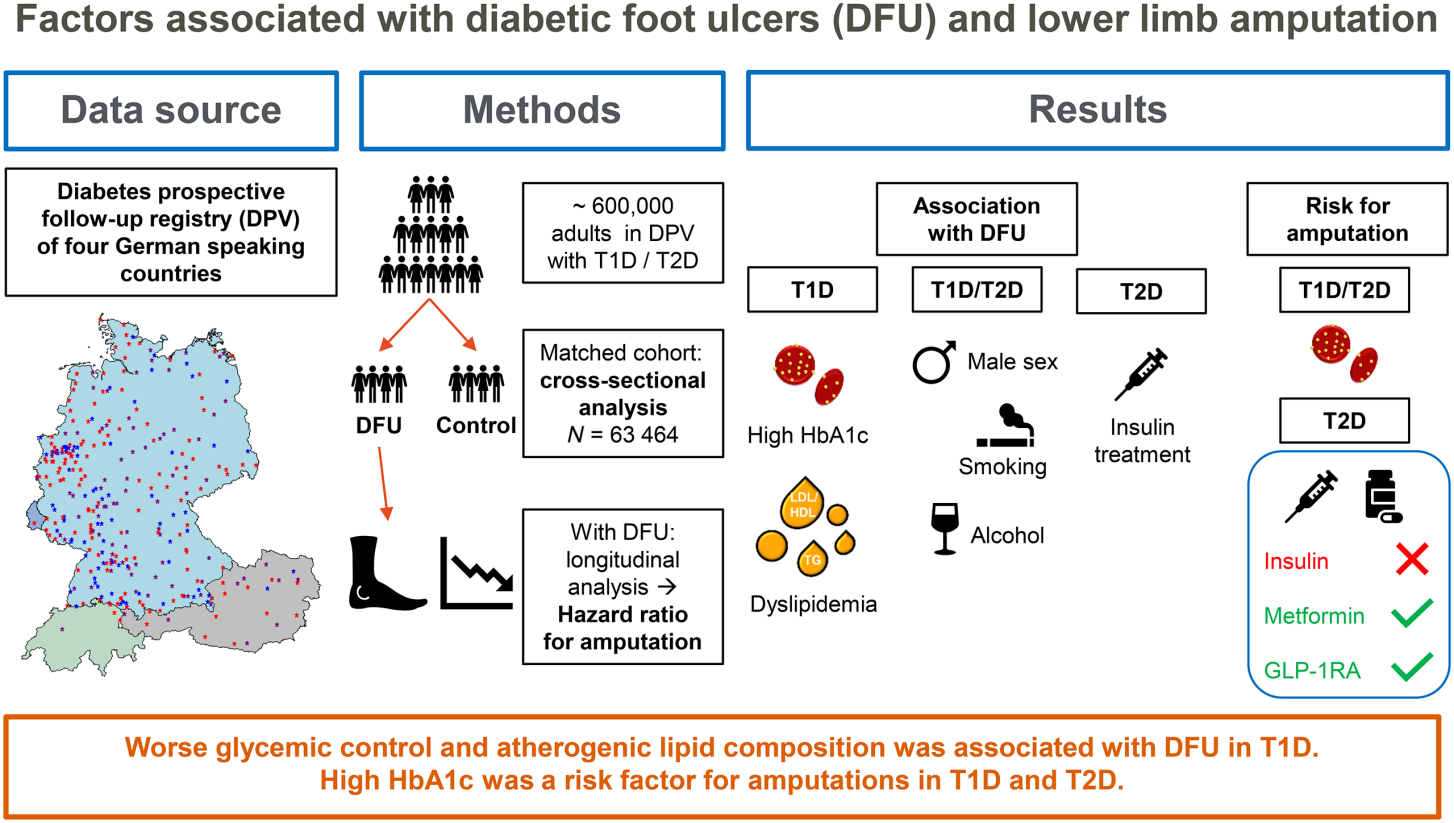

## Graphical abstract

Highlights

- Poor glycemic control and high lipid levels were associated more closely with diabetic foot ulcers in type 1 compared to type 2 diabetes.
- Gender differences regarding the association of diabetic foot ulcers with metabolic outcomes were more pronounced in type 1 diabetes.
- Smoking and alcohol consumption might be important in developing foot ulcers but play a minor role in prevention of amputations.
- The role of oral hypoglycemic medication in development of diabetic foot ulcers should be further analyzed in type 2 diabetes.

## 1 INTRODUCTION

Diabetic foot ulcers (DFU) are frequent complications in adults with diabetes mellitus. The global prevalence of DFU is estimated to be 6.3% with a slightly lower prevalence in European countries (5.1%).^1^ DFU are caused by peripheral neuropathy in combination with vascular diseases.^2^ The DFU recurrence rate is high with 50% recurrence within 3 years.^3^ DFU often leads to minor (below the ankle) or major (above the ankle) amputations. It is estimated that within 1 year about 20% and 10% of adults with DFU require minor and major amputations, respectively.^4^ Moreover, a major amputation increases the mortality rate by 50% within the 5 years.^5^

Male sex, tall height, high hemoglobin A1c (HbA1c) and cigarettes smoking are widely recognized risk factors for DFU.^6^, ^7^, ^8^ Additionally, diabetes complications such as neuropathy, peripheral artery disease (PAD) and osteomyelitis are well‐established risk factors for DFU.^7^, ^9^, ^10^ However, studies have reported conflicting results for clinical variables such as body mass index (BMI), hypertension, lipid levels and alcohol consumption, and its association with DFU.^11^, ^12^ Although these factors may be improved by treatment strategies in at‐risk individuals, some of the better confirmed risk factors such as height and sex are not amenable to intervention. Previous studies were limited by analysis of only a few risk factors and/or small sample size.

Considering literature gap, this study aimed to identify risk factors associated with DFU and lower limb amputations using real‐world data from a large cohort of adults with type 1 (T1D) and type 2 diabetes (T2D).

## 2 MATERIALS AND METHODS

### 2.1 Data collection

The current analysis is based on the prospective, multicenter diabetes patient registry (DPV [Diabetes‐Patienten‐Verlaufsdokumentation]), which is a standardized electronic health record developed at the Institute of Epidemiology and Medical Biometry, Ulm University, Germany. The DPV registry provides long‐term real‐world data on diabetes treatment and outcome from more than 500 centers in Germany, Austria, Switzerland, and Luxembourg. The DPV initiative and the analysis of pseudonymized data were approved by the Ethics Committee of Ulm University (approval number: 314/21) as well as by local review boards. The biannually transmitted data are checked for inconsistency or implausibility and reported back to the respective centers for correction, if necessary.

### 2.2 Design and participants

The documentation of patient data is prospective in the DPV registry and these data were retrospectively analyzed. All adults (age ≥ 18 years) with a diagnosis of T1D or T2D in the DPV registry were included if they had documented data between 2000 and 2021 and a disease duration of ≥1 year. Individuals with a documented diagnosis of DFU or lower limb amputation before the year 2000, before the age of 18 years, within the first year after their diabetes manifestation, or at an unknown date were excluded from this analysis. Additionally, individuals with a Wagner grade of 0, indicating pre‐ or postulcerative lesions (*N* = 17 165, 4.6%), were excluded as they could neither be appropriately allocated to the DFU group nor to the control group (Figure S1). All remaining individuals were allocated to one of four groups (separately for T1D and T2D): (a) no DFU and no lower limb amputation, (b) DFU without any lower limb amputation, (c) DFU and a minor amputation, and (d) DFU and a major amputation. For this stratification, lower limb amputations comprised all amputations at the visit of the first documentation of DFU and thereafter. Amputations were classified as minor if the amputation was performed below the ankle and as major if the amputation was conducted above the ankle.

For group (a) without DFU, aggregated data from the most recent treatment year of each individual were analyzed. For individuals in the other three groups, the year before the first diagnosis of DFU was analyzed for cross‐sectional comparisons to people without DFU. For these individuals, the visits following their first documentation of DFU were additionally analyzed with respect to amputations (longitudinal analysis).

### 2.3 Propensity score matching

A total of 359 460 individuals fulfilled the inclusion criteria (31 801 with DFU). To control for age, sex, diabetes duration, and treatment year, the individuals with DFU were matched one to one to the control group without DFU regarding these variables, separately for T1D and T2D. The variables sex and treatment year were matched exactly. Age and diabetes duration were included continuously as well as categorized (T1D: age 18 < 25, 25– <50, 50+ years, diabetes duration 1–10, >10–30, >30 years; T2D: age 18– <60, 60– <80, 80+ years, diabetes duration 1–5, >5–10, >10–20, >20 years) into the nonparsimonious propensity score. One‐to‐one matching was conducted using a greedy‐matching algorithm with a caliper width of 0.2. Standardized differences were assessed before and after matching to evaluate balancing of covariates between the matched cohorts (Table S1). A standardized difference of <10% for a baseline covariate reveals a negligible imbalance.^13^ For 31 732 individuals with DFU a matching partner could be allocated leading to a final cohort of 63 464 adults with type 1 diabetes (*N* = 5352) or type 2 diabetes (*N* = 58 112).

### 2.4 Data management

The diagnosis and classification of diabetes types were conducted by the diabetologists at the participating centers according to the clinical presentation of the people with a diabetes manifestation and lab results, based on German guidelines.^14^ Anthropometric measurements were performed in the local centers according to in‐house protocols. The BMI was calculated as weight in kilograms divided by height in meters squared. Lipid values were converted to mg/dL. Total cholesterol (TC), low‐density lipoprotein (LDL), high‐density lipoprotein (HDL), and triglycerides (TG) were analyzed. Non‐HDL was calculated through the following formula: TC–HDL. Additionally, the ratio of TC/HDL and LDL/HDL was calculated. HbA1c values were standardized to the Diabetes Control and Complications Trial reference range of 4.05%–6.05% (20.7–42.6 mmol/mol) using the multiple of the mean transformation method to account for different laboratory methods.^15^, ^16^ Hypertension was defined as systolic blood pressure (BP) ≥140 mm Hg or diastolic blood pressure ≥90 mm Hg. Metabolic syndrome was defined per the International Diabetes Federation Consensus Statement^17^ as obesity (BMI ≥30 kg/m^2^, because waist circumference was not available in most individuals) and one of the following: TG >150 mg/dL, HDL <40 mg/dL (males) or <50 mg/dL (females), lipid‐lowering medication, systolic BP ≥130 mm Hg or diastolic BP ≥85 mm Hg, or antihypertensive medication. The third criterion of elevated fasting plasma glucose was present in all participants through the documentation of diabetes. Smoking was defined as smoking at least one cigarette per day (yes/no). Alcohol abuse was considered as a daily alcohol consumption of >24 g in men and >12 g in women.^18^ Missing data on smoking behavior were not used for analysis, whereas no documentation of critical alcohol intake at any visit was classified as no alcohol abuse. Smoking behavior, alcohol abuse as well as documented diagnoses of neuropathy, PAD, and retinopathy^19^ according to guideline‐based in‐house evaluations of the clinicians were aggregated over the whole documentation period (for adults with DFU only before the first documentation of DFU). Nephropathy was defined as estimated glomerular filtration rate <60 (mL/min/1.73^2^) according to the Chronic Kidney Disease Epidemiology Collaboration formula^20^ or as documented microalbuminuria or if renal transplantation or a history of dialysis was documented. For identification and classification of the severity of DFU Wagner grades were used as this classification system is still recommended in the most recent guideline on diabetic foot from the German Diabetes Association and therefore was used by the centers participating in this study during foot examinations and is included in the DPV documentation software.^21^ Wagner grades range from 0 to 5 indicating no current ulcer, but pre‐ or postulcerative lesions (Wagner 0), superficial ulcer (Wagner 1), deep ulcer without abscess or bone involvement (Wagner 2), ulcer with abscess and bone involvement (Wagner 3), localized ulcer with gangrene (Wagner 4), and extensive ulcer with gangrene (Wagner 5). Wagner grades of 1 or higher were defined as DFU. Individuals with Wagner 0 were excluded. For each individual the highest documented classification at time of first occurrence of DFU was considered in individuals with DFU. In individuals without DFU the documented foot examination within the most recent treatment year was used for identification of those with Wagner 0. If one of the two matching partners had missing values on an outcome variable, data from both matching partners were excluded from the analysis on this variable to avoid bias through asymmetry of missing data between people with and without DFU.

### 2.5 Statistical analysis

All statistical analyses were generated using SAS (Statistical Analysis Software, SAS Institute Inc., Cary, NC, USA) Version 9.4, Built M7, on a Windows Server 2016 mainframe. For all analyses a two‐sided *p* value of <.05 was considered as significant. Descriptive statistics were performed for all individuals before matching and stratified by DFU and amputation separately for T1D and T2D (Table 1). The results are shown as median with quartiles for continuous variables and as proportions for binary variables. *p* values were calculated using the chi‐square test for categorial variables and the Wilcoxon's rank sum test for continuous variables and adjusted for multiple comparisons, separately for T1D and T2D. Besides the unadjusted proportions of people with DFU or amputations by diabetes type among the unmatched cohort, odds ratios (OR) were calculated using logistic regression models, adjusted for age categories (18– <40, 40– <60, 60+ years), sex, diabetes duration categories (1–10, >10–25, >25 years), calendar year categorized (2000–2005, 2006–2010, 2011–2021), smoking history, and alcohol abuse to compare the risk for DFU and amputation of individuals with T1D and T2D. Cross‐sectional comparisons of the matched cohort with DFU vs the control group were conducted irrespective of DFU with or without amputations, both for the whole group and stratified by sex. *p* values were calculated in the same way as within the descriptive analysis and adjustment for multiple comparisons was implemented within each subgroup. The risk for amputation following the first documentation of DFU was analyzed with Cox regression to calculate hazard ratios (HRs). Individuals with DFU and at least 100 days of follow‐up (first documentation of DFU was considered as baseline), but without an amputation within these first 100 days were included. Therefore, 1107 adults with T1D and 8586 adults with T2D were selected for longitudinal analysis (Figure S1). Due to the reduced number of individuals included in this analysis, minor and major amputations were pooled as outcome variable. HRs were calculated unadjusted and adjusted for sex, age categories, diabetes duration, and calendar year at baseline. Age groups for this analysis differed from age groups used for matching in T1D, as this follow‐up cohort was considerably older (age groups for follow‐up analysis in T1D: 18– <40, 40– <60, 60+ years). We additionally conducted a “complete model” analysis with all relevant variables included in one model. Individuals were censored at the time of last contact if an amputation was never conducted.

**TABLE 1:** Characteristics of unmatched cohort stratified by diabetic foot ulcer and amputation.

Variable	T1D					T2D				
	All	No DFU	DFU only	DFU + Min	DFU + Maj	All	No DFU	DFU only	DFU + Min	DFU + Maj
	*N* = 54 131	*N* = 51 404	*N* = 1687	*N* = 704	*N* = 336	*N* = 305 329	*N* = 276 2	*N* = 18 457	*N* = 7012	*N* = 3605
Sex (% male)	52.8	52.4	56.4 **	71.0 ***	66.7 ***	52.3	51.2	60.3 ***	71.7 ***	69.5 ***
Age (years)	32.8 [19.6–53.6]	30.8 [19.4–51.9]	59.5 *** [47.6–69.7]	57.8 *** [48.2–68.8]	58.5 *** [48.9–67.7]	71.0 [61.4–78.8]	70.9 [61.2–78.8]	72.1 *** [63.5–79.2]	70.3 * [62.6–77.2]	71.0 [63.2–77.8]
Diabetes duration (years)	13.2 [7.3–23.2]	12.8 [7.2–22.1]	23.0 *** [13.5–35.3]	30.0 *** [19.3–39.8]	31.4 *** [19.7–41.4]	10.2 [5.4–16.7]	10.1 [5.2–16.4]	11.9 *** [6.9–19.6]	14.3 *** [8.6–21.1]	13.2 *** [8.2–20.5]
Treatment year	2015 [2009–19]	2015 [2010–19]	2010 *** [2005–15]	2011 *** [2006–15]	2010 *** [2006–15]	2013 [2009–17]	2014 [2009–17]	2012 *** [2008–16]	2011 *** [2007–15]	2010 *** [2006–14]
Height (cm)	172 [165–179]	172 [165–180]	172 [164–179]	175 *** [168–181]	173 [166–180]	169 [162–176]	168 [162–175]	171 *** [164–178]	173 *** [167–180]	172 *** [165–178]
Weight (kg)	73.5 [64.5–84.3]	73.3 [64.4–84.0]	77.5 *** [67.5–90.0]	77.1 *** [67.6–90.0]	75.0 [62.0–89.4]	85.0 [73.8–99.5]	85.0 [73.5–99.0]	87.0 *** [75.0–102.7]	87.5 *** [76.0–101.2]	83.4 *** [72.0–97.0]
BMI (kg/m^2^)	24.6 [22.1–27.8]	24.5 [22.1–27.7]	26.2 *** [23.0–29.8]	25.0 ** [22.5–28.9]	24.7 [21.8–29.1]	29.8 [26.2–34.3]	29.8 [26.2–34.3]	29.7 [26.0–34.4]	29.2 *** [25.8–33.2]	28.3 *** [24.8–32.3]
HbA1c (%)	7.7 [6.9–8.9]	7.7 [6.9–8.9]	7.7 [6.8–8.9]	7.9 * [7.0–9.2]	7.8 [6.9–8.9]	7.2 [6.3–8.4]	7.2 [6.3–8.4]	7.2 [6.3–8.3]	7.3 *** [6.4–8.5]	7.2 [6.3–8.5]
HbA1c (mmol/mol)	61 [52–74]	61 [52–74]	60 [50–74]	63 * [53–77]	61 [51–74]	55 [46–68]	55 [46–68]	55 [46–67]	56 *** [46–70]	55 [46–69]
TG (mg/dl)	101 [71–151]	100 [71–149]	116 *** [84–171]	114 *** [85–167]	125 *** [89–186]	153 [108–221]	154 [109–222]	144 *** [104–205]	140 *** [100–200]	151 [108–221]
On insulin (%)						58.7	57.5	65.8 ***	76.6 ***	79.4 ***
Pump use (%)	31.7	32.7	13.3 ***	10.5 ***	14.6 ***					
Neuropathy (%)	24.9	22.5	73.9 ***	68.0 ***	61.0 ***	38.5	36.8	64.5 ***	57.0 ***	49.1 ***
Retinopathy (%)	17.1	15.6	35.0 ***	48.2 ***	54.7 ***	12.0	10.9	18.5 ***	29.5 ***	28.6 ***
Nephropathy (%)	20.3	18.7	40.9 ***	57.8 ***	55.2 ***	50.0	49.1	55.4 ***	62.6 ***	62.1 ***
PAD (%)	5.3	3.6	34.7 ***	41.1 ***	46.4 ***	12.5	9.3	41.3 ***	45.2 ***	50.5 ***
Hypertension (%)	25.0	24.3	37.8 ***	42.0 ***	34.5 ***	43.4	43.4	43.6	42.6	43.7
Dyslipidemia (%)	44.3	43.6	56.3 ***	51.0 **	60.7 ***	66.4	66.6	64.1 ***	64.1 ***	69.2 *
Smoking (%)	29.0	29.0	28.3	28.7	31.4	14.5	14.5	14.1	15.1	16.8 **
Alcohol abuse (%)	4.2	4.0	8.3 ***	8.4 ***	8.6 ***	3.7	3.4	6.3 ***	6.8 ***	6.0 ***

*Note*: Characteristics at the most recent treatment year or the year before the first documentation of diabetic foot ulcer. Data are shown as median with interquartile range or as %. All *p* values were calculated for the comparison to the control group without any diabetic foot ulcers and adjusted for multiple comparisons, separately for T1D and T2D.

Abbreviations: BMI, body mass index; DFU, diabetic foot ulcer; DFU‐Min, diabetic foot ulcer with minor amputation; DFU‐Maj, diabetic foot ulcer with major amputation; DFU only, diabetic foot ulcer without amputation; HbA1c, glycated hemoglobin; No DFU, control group without any diabetic foot ulcer; PAD, peripheral artery disease; TG, triglycerides; T1D, type 1 diabetes; T2D, type 2 diabetes.

* *p* < .05;

** *p* < .01;

*** *p* < .001.

## 3 RESULTS

### 3.1 Description of unmatched cohort

Among all individuals in the unmatched cohort (*N* = 359 460), the proportion of males was 53% in T1D and 52% in T2D. Median age with interquartile range was considerably lower in T1D (32.8 [19.6–53.6] years) than in T2D (71.0 [61.4–78.8] years), but diabetes duration was longer (T1D: 13.2 [7.3–23.2] years; T2D: 10.2 [5.4–16.7] years). For both diabetes types, individuals with DFU were more often male, were older, had longer diabetes duration, and the analyzed calendar year been earlier. Further characteristics of the unmatched cohort are presented in Table 1 and standardized differences of matching variables before and after matching are shown in Table S1. All matching variables could be equalized sufficiently as all standardized differences were ≤1% after the matching procedure.

Besides individuals with Wagner grade 0, who were excluded from the analysis, about one half of the remaining individuals had a documented Wagner grade. Of those with a documented foot examination, 88.5% were classified as healthy (no signs of DFU) and 5.0%, 3.0%, 2.2%, 1.2%, and 0.1% had the Wagner grades 1–5.

### 3.2 Proportion with DFU

The unadjusted proportion of people with DFU was 5.0% in T1D and 9.5% in T2D. The adjusted OR with 95% confidence limits for DFU for T2D vs T1D was 1.22 [1.16–1.29], *p* < .001. Among individuals with DFU, the unadjusted proportion with amputations in the subsequent course of their disease was 36.5% in T1D and 38.1% in T2D representing an adjusted OR for amputations among adults with T2D of 1.17 [1.06–1.30] (*p* = .003) compared to T1D.

### 3.3 Cross‐sectional comparisons

Within the matched cohort with T1D, the factors associated with DFU were higher body height (*p* = .013), increased HbA1c (*p* < .001), triglycerides as well as the ratio of TC/HDL or LDL/HDL (all *p* < .01), smoking history (*p* = .007), and multiple subcutaneous daily injections with insulin pens (*p* < .001). The results regarding triglycerides levels were similar even with additional adjustment for HbA1c, type of insulin administration (injection vs pump use) and prescription of lipid‐lowering medication. No clear evidence was observable for blood pressure, but the proportion treated with antihypertensive medication was higher in individuals with DFU (*p* < .001, Table 2). In women, BMI, metabolic syndrome, and lipid levels, especially TG and the ratios of TC/HDL or LDL/HDL, were related to DFU, whereas in men HbA1c and a smoking history were more prominent (Table 2). In adults with T2D, alcohol abuse, a smoking history, and insulin therapy (all *p* < .001) were related to DFU, whereas HbA1c did not differ and lipid levels as well as the proportion with hypertension were lower in individuals with DFU. Similarly to T1D, the proportion treated with antihypertensive medication was higher (*p* < .001) in individuals with DFU (Table 3). For both diabetes types the proportion with documented neuropathy, PAD and nephropathy was significantly increased (all *p* < .001) whereas the increase in retinopathy was significant only for T2D individuals with DFU (Tables 2 and 3).

**TABLE 2:** Main outcomes of matched cohort with T1D stratified by sex.

Variable	All		Female		Male	
	*N* = 5352		*N* = 2042		*N* = 3310	
	No DFU	With DFU	No DFU	With DFU	No DFU	With DFU
Height (cm) *N* (all) = 4986	172 [165–178]	173* [165–180]	164 [160‐169]	165 [160–169]	176 [172–181]	178*** [173–183]
Body mass index (kg/m^2^) *N* (all) = 4986	25.5 [23.0–28.7]	25.7 [22.7–29.4]	25.3 [22.4–28.6]	26.0 ** [22.6–30.5]	25.8 [23.4–28.7]	25.6 [22.8–29.0]
HbA1c (%) *N* (all) = 5352	7.5 [6.8–8.5]	7.8*** [6.9–9.0]	7.6 [6.9–8.5]	7.7 * [6.9–9.0]	7.3 [6.7–8.5]	7.8*** [6.9–9.0]
HbA1c (mmol/molv *N* (all) = 5352	59 [50–69]	61*** [51–75]	59 [52‐69]	61* [51–75]	58 [50‐69]	62*** [51–75]
TC (mg/dL) *N* (all) = 2354	192 [162–220]	190 [158–219]	200 [174–225]	201 [174–231]	188 [157–215]	179 [151–211]
HDL (mg/dL) *N* (all) = 2354	58 [46–74]	52*** [41–66]	67 [52‐82]	58*** [46–72]	54 [43‐69]	49*** [39–61]
LDL (mg/dL) *N* (all) = 2354	104 [80–130]	99 [75–126]	107 [82–134]	106 [79–133]	102 [79–127]	97 [74–123]
TC/HDL (mg/dL) *N* (all) = 2354	3.19 [2.57–4.07]	3.54*** [2.80–4.54]	2.95 [2.43–3.63]	3.41*** [2.66–4.44]	3.34 [2.67–4.30]	3.64*** [2.86–4.64]
LDL/HDL (mg/dL) *N* (all) = 2354	1.74 [1.23–2.44]	1.94** [1.29–2.71]	1.58 [1.13–2.19]	1.79* [1.17–2.47]	1.88 [1.30–2.68]	2.05 [1.38–2.79]
Triglycerides (mg/dL) *N* (all) = 2980	101 [74–146]	116*** [84–170]	94 [72‐141]	116*** [81–172]	104 [76‐151]	115*** [85–170]
Hypertension (%) *N* (all) = 5002	36.6	38.3	35.4	36.8	37.3	39.1
Antihypertensives (%) *N* (all) = 5352	47.1	55.4***	46.4	58.2***	47.6	53.7**
Metabolic syndrome (%) *N* (all) = 4982	20.4	25.3***	21.4	31.0***	22.9	24.3
Smoking (%) *N* (all) = 3142	24.1	29.4**	20.6	23.1	26.3	33.3**
Alcohol abuse (%) *N* (all) = 5352	6.5	8.4	3.9	4.5	8.2	10.8
Pump use (%) *N* (all) = 5352	24.5	12.9***	27.1	15.3***	22.8	11.5***
Neuropathy (%) *N* (all) = 5352	46.7	70.3***	45.3	70.0***	47.6	70.5***
PAD (%) *N* (all) = 5352	9.0	37.6***	7.3	37.6***	10.0	37.5***
Nephropathy (%) *N* (all) = 5352	32.7	46.7***	35.2	47.8***	31.1	46.0***
Retinopathy (%) *N* (all) = 1532	36.2	41.1	31.8	40.5	39.0	41.5

*Note*: Data are shown as median with interquartile range or as %.

Abbreviations: DFU, diabetic foot ulcer; HbA1c, glycated hemoglobin; HDL, high‐density lipoprotein; LDL, low‐density lipoprotein; No DFU, control group without any diabetic foot ulcer; PAD, peripheral artery disease; TC, total cholesterol; T1D, type 1 diabetes.

* *p* < .05;

** *p* < .01;

*** *p* < .001.

**TABLE 3:** Main outcomes of matched cohort with T2D stratified by sex.

Variable	All		Female		Male	
	*N* = 58 112		*N* = 20 806		*N* = 37 306	
	No DFU	With DFU	No DFU	With DFU	No DFU	With DFU
Height (cm) *N* (all) = 50 284	170 [164–176]	172 *** [165–178]	161 [158‐166]	163 *** [159–168]	174 [170‐179]	176 *** [171–181]
Body mass index (kg/m^2^) *N* (all) = 50 284	29.4 [26.1–33.7]	29.4 [25.9–34.0]	29.8 [25.9–34.6]	29.8 [25.7–34.8]	29.4 [26.1–33.3]	29.3 [25.9–33.5]
HbA1c (%) *N* (all) = 58 112	7.2 [6.4–8.4]	7.2 [6.3–8.4]	7.2 [6.4–8.4]	7.2 [6.4–8.3]	7.2 [6.4–8.4]	7.2 [6.3–8.4]
HbA1c (mmol/mol) *N* (all) = 58 112	55 [46–68]	55 [46–68]	55 [46–68]	55 [46–67]	56 [46–68]	55 [46–68]
TC (mg/dL) *N* (all) = 22 324	180 [149–214]	174 *** [143–208]	192 [160‐226]	186 *** [154–221]	173 [143‐207]	167 *** [138–199]
HDL (mg/dL) *N* (all) = 22 324	43 [35–53]	41 *** [34–51]	47 [38‐58]	45 *** [37–56]	41 [34‐50]	40 *** [32–48]
LDL (mg/dL) *N* (all) = 22 324	104 [77–132]	100 *** [75–127]	110 [83‐139]	106 ** [81–135]	100 [75‐128]	96 *** [72–123]
TC/HDL (mg/dL) *N* (all) = 22 324	4.11 [3.23–5.23]	4.17 [3.28–5.29]	4.03 [3.16–5.13]	4.04 [3.21–5.18]	4.16 [3.28–5.27]	4.21 [3.33–5.33]
LDL/HDL (mg/dL) *N* (all) = 22 324	2.38 [1.71–3.23]	2.41 [1.74–3.24]	2.33 [1.67–3.17]	2.35 [1.67–3.17]	2.41 [1.73–3.26]	2.45 [1.78–3.29]
Triglycerides (mg/dL) *N* (all) = 28 212	151 [106–217]	145 *** [103–207]	155 [111‐218]	152 [108–216]	148 [104–215]	142 *** [101–201]
Hypertension (%) *N* (all) = 53 580	44.5	43.3 *	45.3	42.7 **	44.0	43.6
Antihypertensives (%) *N* (all) = 58 112	56.2	63.9 ***	57.4	65.4 ***	55.6	63.1 ***
Metabolic syndrome (%) *N* (all) = 50 052	48.2	49.8 **	50.8	52.5	46.7	48.4 *
Smoking (%) *N* (all) = 29 172	13.1	14.7 ***	7.8	9.7 **	16.1	17.5
Alcohol abuse (%) *N* (all) = 58 112	3.8	6.4 ***	1.5	2.5 ***	5.1	8.6 ***
Insulin therapy (%) *N* (all) = 58 112	63.3	70.1 ***	63.7	70.2 ***	63.2	70.0 ***
Neuropathy (%) *N* (all) = 58 112	39.4	60.8 ***	38.2	61.5 ***	40.1	60.4 ***
PAD (%) *N* (all) = 58 112	10.8	43.4 ***	10.3	41.7 ***	11.1	44.4 ***
Nephropathy (%) *N* (all) = 58 112	50.7	58.2 ***	58.6	65.4 ***	46.3	54.2 ***
Retinopathy (%) *N* (all) = 8460	14.6	23.2 ***	13.8	24.1 ***	15.1	22.7 ***

*Note*: Data are shown as median with interquartile range or as %.

Abbreviations: DFU, diabetic foot ulcer; HbA1c, glycated hemoglobin; HDL, high‐density lipoprotein; LDL, low‐density lipoprotein; No DFU, control group without any diabetic foot ulcer; PAD, peripheral artery disease; TC, total cholesterol; T2D, type 2 diabetes.

* *p* < .05;

** *p* < .01;

*** *p* < .001.

### 3.4 Longitudinal analysis

**FIGURE 1:**
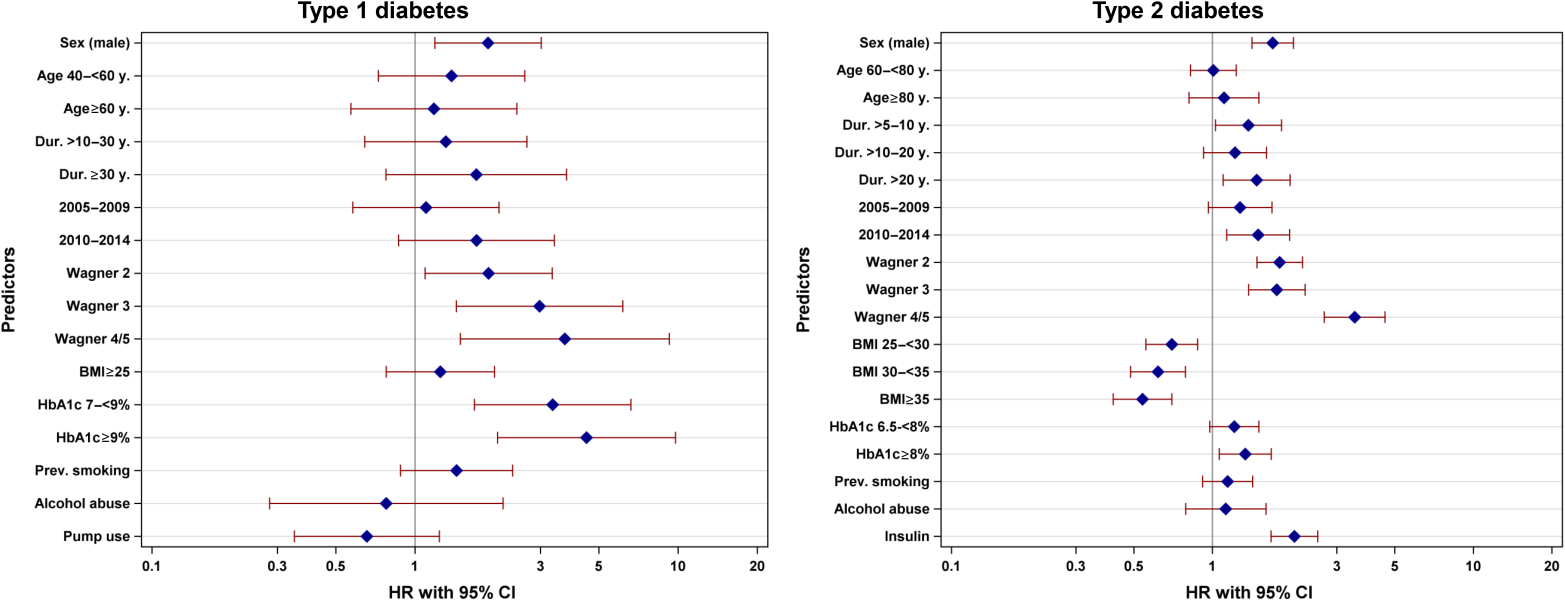
Hazard ratios for the risk of amputation in individuals with diabetic foot ulcers. All individuals were followed for at least 100 days and initial amputations within the first 100 days after the first documentation of diabetic foot ulcer were excluded. Hazard ratios were calculated in a complete model including all shown parameters. BMI, body mass index; CI, confidence interval; DFU, diabetic foot ulcers; HbA1c, glycated hemoglobin; HR, hazard ratio.

Cox regression analyses of the 9693 individuals with DFU and sufficient follow‐up data revealed that in T1D, male sex, long diabetes duration, high Wagner grades, and high HbA1c were the strongest predictors for future amputations (Table 4). These results remained significant even in the “complete model” analysis with the exception of diabetes duration (Figure 1). In T2D, the same predictors could be observed, but high BMI was associated with lower HRs for future amputation (Table 4). Again, these results remained more or less stable in the “complete model” except for diabetes duration and HbA1c to some extent (Figure 1). Individuals on insulin had higher HRs whereas individuals on metformin or GLP‐1 receptor agonists (GLP‐1RA) had lower HRs (Table 4), but this effect vanished partly combining all medications in one model (Figure S2). The crude HRs for those using sodium glucose transporter 2 inhibitors (SGLT2i) were elevated, but the statistical significance vanished with adjustment for age, sex, diabetes duration, and treatment year in both groups (with additional insulin therapy and those without insulin treatment). The crude HRs were higher in the group without insulin therapy (Table 4). Smoking and alcohol abuse did not reveal significantly higher HRs for amputations in both T1D and T2D. Data from the “complete model” analysis on most important predictors are shown in Figure 1 and all analyzed HRs, crude and adjusted, are presented in Table 4.

**TABLE 4:** Crude and adjusted hazard ratios for amputations.

Predictive variable	T1D		T2D	
	Crude HR	Adjusted HR	Crude HR	Adjusted HR
Sex (male)	2.4 [1.6–3.5], *p* < .001	2.2 [1.5–3.2], *p* < .001	2.0 [1.7–2.3], *p* < .001	1.7 [1.5–2.0], *p* < .001
Age				
18–39 years	ref.	ref.		
40–59 years	1.3 [0.8–2.2], *p* = .290	1.3 [0.8–2.1], *p* = .360		
60+ years	0.8 [0.4–1.3], *p* = .316	0.8 [0.4–1.3], *p* = .364		
18–59 years			ref.	ref.
60–79 years			1.0 [0.9–1.1], *p* = .782	0.9 [0.8–1.1], *p* = .322
80+ years			1.1 [0.9–1.4], *p* = .369	1.1 [0.8–1.3], *p* = .639
Diabetes duration				
1–10 years	ref.	ref.		
11–30 years	2.3 [1.3–4.3], *p* = .007	2.0 [1.1–3.8], *p* = .024		
31+ years	3.6 [1.9–6.7], *p* < .001	2.9 [1.5–5.6], *p* = .001		
1–5 years			ref.	ref.
6–10 years			1.5 [1.2–1.9], *p* < .001	1.5 [1.2–1.9], *p* < .001
11–20 years			1.8 [1.5–2.2], *p* < .001	1.7 [1.4–2.1], *p* < .001
21+ years			2.4 [1.9–2.9], *p* < .001	2.2 [1.8–2.7], *p* < .001
Calendar year				
2000–2005	ref.	ref.	ref.	ref.
2006–2010	1.5 [0.9–2.4], *p* = .125	1.2 [0.8–2.0], *p* = .374	1.7 [1.4–2.1], *p* < .001	1.6 [1.3–1.9], *p* < .001
2011–2021	4.0 [2.6–6.3], *p* < .001	2.9 [1.8–4.6], *p* < .001	2.3 [1.9–2.8], *p* < .001	1.9 [1.6–2.3], *p* < .001
Wagner‐grade				
1	ref.	ref.	ref.	ref.
2	4.3 [2.7–6.7], *p* < .001	3.0 [1.8–4.9], *p* < .001	3.2 [2.7–3.8], *p* < .001	2.6 [2.2–3.1], *p* < .001
3	7.8 [4.5–13.4], *p* < .001	4.5 [2.5–8.1], *p* < .001	4.0 [3.3–4.8], *p* < .001	3.0 [2.4–3.6], *p* < .001
4/5	9.0 [4.2–19.3], *p* < .001	5.2 [2.3–11.7], *p* < .001	6.9 [5.5–8.6], *p* < .001	5.2 [4.2–6.6], *p* < .001
BMI				
<25	ref.	ref.		
≥25	0.7 [0.5–1.1], *p* = .102	0.9 [0.6–1.3], *p* = .605		
<25			ref.	ref.
25– <30			0.7 [0.6–0.8], *p* < .001	0.7 [0.5–0.8], *p* < .001
30– <35			0.7 [0.5–0.8], *p* < .001	0.7 [0.6–0.8], *p* < .001
≥35			0.5 [0.4–0.6], *p* < .001	0.5 [0.4–0.6], *p* < .001
HbA1c				
<7%	ref.	ref.		
7– <9%	3.2 [2.0–5.2], *p* < .001	2.6 [1.6–4.2], *p* = .001		
≥9%	4.6 [2.7–7.8], *p* < .001	3.4 [1.9–6.0], *p* < .001		
<6.5%			ref.	ref.
6.5– <8%			1.5 [1.3–1.7], *p* < .001	1.3 [1.1–1.5], *p* = .002
≥8%			1.9 [1.6–2.2], *p* < .001	1.6 [1.3–1.8], *p* < .001
Smoking	1.6 [1.1–2.5], *p* = .017	1.4 [0.9–2.1], *p* = .136	1.3 [1.1–1.6], *p* = .010	1.2 [1.0–1.5], *p* = .061
Alcohol	0.8 [0.3–2.0], *p* = .664	0.7 [0.3–1.8], *p* = . 514	0.9 [0.7–1.3], *p* = .673	0.8 [0.6–1.2], *p* = .291
Hypertension	1.2 [0.8–1.6], *p* = .402	1.3 [0.9–1.9], *p* = .132	0.9 [0.8–1.0], *p* = .013	0.9 [0.8–1.0], *p* = .046
Dyslipidemia	1.0 [0.7–1.5], *p* = .901	1.2 [0.8–1.7], *p* = .421	0.8 [0.7–1.0], *p* = .018	0.9 [0.8–1.1], *p* = .268
TG > 150 mg/dL	1.3 [0.9–1.9], *p* = .248	1.3 [0.9–1.9], *p* = .249	0.8 [0.7–0.9], *p* = .004	0.9 [0.7–1.0], *p* = .031
Hypertensive medication	0.9 [0.7–1.3], *p* = .756	0.9 [0.6–1.3], *p* = .608	1.2 [1.0–1.3], *p* = .023	1.1 [1.0–1.2], *p* = .202
Lipid‐lowering medication	0.9 [0.6–1.3], *p* = .610	0.9 [0.6–1.3], *p* = .498	1.0 [0.9–1.1], *p* = .682	0.9 [0.8–1.0], *p* = .025
Pump use	0.8 [0.5–1.3], *p* = .354	0.6 [0.4–1.0], *p* = .053		
Insulin use			2.2 [1.9–2.5], *p* < .001	2.1 [1.8–2.4], *p* < .001
Metformin			0.6 [0.5–0.6], *p* < .001	0.6 [0.5–0.7], *p* < .001
Excluding insulin^a^			0.6 [0.5–0.8], *p* < .001	0.8 [0.6–1.0], *p* = .027
GLP‐1RA			0.7 [0.5–1.1], *p* = .133	0.6 [0.4–0.9], *p* = .015
Excluding insulin^a^			0.3 [0.1–1.3], *p* = .100	0.2 [0.1–0.9], *p* = .037
Sulphonylurea			0.5 [0.4–0.6], *p* < .001	0.6 [0.5–0.7], *p* < .001
Excluding insulin^a^			0.9 [0.7–1.2], *p* = .422	1.1 [0.9–1.5], *p* = .344
SGLT2i			1.5 [1.0–2.3], *p* = .038	1.1 [0.8–1.7], *p* = .543
Excluding insulin^a^			2.4 [1.3–4.6], *p* = .0061.7	1.8 [0.9–3.4], *p* = .080
DPP4i			1.3 [1.1–1.6], *p* = .002	1.0 [0.9–1.2], *p* = .785
Excluding insulin^a^			1.7 [1.2–2.3], *p* = .001	1.3 [1.0–1.9], *p* = .092

*Note*: Shown are HRs for minor or major amputations, crude and adjusted for age, sex, diabetes duration, and treatment years.

Abbreviations: BMI, body mass index; DPP4i, dipeptidyl peptidase‐4 inhibitors; GLP‐1RA, GLP1‐receptor agonists; HbA1c, glycated hemoglobin; HR, hazard ratio; SGLT2i, sodium‐glucose cotransporter‐2 inhibitors; TG, triglycerides; T1D, type 1 diabetes; T2D, type 2 diabetes.

^**a**^ Individuals on insulin were excluded for this analysis.

## 4 DISCUSSION

In the present study, we found slightly higher odds for DFU and amputations in adults with T2D compared to T1D. In adults with T1D, high HbA1c, high lipid levels, and multiple subcutaneous daily injections with insulin pens (compared to insulin pump) were associated with DFU. In adults with T2D, a smoking history, alcohol abuse, and insulin treatment were related to DFU. The risk for amputations among those with DFU was higher in adults with high HbA1c, but smoking and alcohol abuse did not reach statistical significance. Insulin treatment was associated with higher risks for amputations, and GLP‐1RA and metformin were associated with lower risks for amputation.

According to a meta‐analysis from 2016, the prevalence of DFU was estimated to be 6.3% globally, and 5.1% in Europe.^1^ The prevalence of DFU in Germany was 6.2% based on insurance data from 2013.^22^ Therefore, the proportion of people with DFU among the DPV data between 2000 and 2021 with 5% in T1D and 9.5% in T2D was approximately within this range. It is worth mentioning that the prevalence of DFU in the present study may be an underestimate as some DFU remain undocumented in the electronic health record. Additionally, we included only DFU with a documented date of diagnosis. This may be a reason for the slightly lower prevalence of DFU in our study compared to an earlier publication from the DPV registry from 2018 where 6.5% individuals with T1D and 9.9% with T2D were documented with DFU at any time.^23^ On the other hand, there are mainly individuals with T2D included in the DPV registry who are treated in participating clinics with qualified diabetologists or endocrinologists and therefore describe a group of individuals with T2D with higher disease burden and potentially a slightly higher rate of foot ulcers compared to prevalence rates based on insurance data. The higher proportion of DFU in T2D than in T1D in our study is in agreement with a few but not all previously published studies.^1^, ^11^

The present study shows that high HbA1c is more strongly associated with DFU in T1D (especially in males) than in T2D, but it is a risk factor for future amputations in both diabetes types. This is in line with most studies^8^, ^10^, ^24^; however, there is one meta‐analysis that did not find an association between HbA1c and DFU.^11^ According to a systematic review, improving glycemic control is one of the four key tasks of multidisciplinary centers to avoid major amputations.^25^ The fact that 94% of studies within this review reported a reduction of major amputations in centers acting according to these key tasks depicts both the importance of HbA1c management and the treatment of individuals with DFU in specialized or multidisciplinary centers. Longer disease duration of adults with T1D compared to T2D and generally higher HbA1c might be reasons for the stronger associations between glycemic control and DFU as this phenomenon could also be detected for other complications and diseases.^26^, ^27^

High BMI and lipid levels as well as the presence of metabolic syndrome were associated with DFU but not with amputations in T1D and this association was stronger for women than for men. Particularly, TG and the ratio of TC/HDL and LDL/HDL seem to be important for the development of foot ulceration. The association of dyslipidemia with DFU in T1D was also observed in an earlier investigation of the DPV data that revealed a higher proportion of DFU in individuals with “double diabetes” (T1D plus metabolic syndrome).^28^ Other studies on lipid levels and T1D are rare, but for combined analyses of both diabetes types triglycerides seem to have the strongest relation to DFU and amputations.^9^ Associations of TG and HDL with DFU were also reported.^29^ According to the DPV data, one could assume that metabolic parameters might be a risk factor only in this specific subgroup and therefore it might be helpful to closely monitor lipid levels in T1D people at higher risk for DFU. Nevertheless, there are studies that report worse lipid composition in individuals with T2D and DFU.^30^, ^31^

In our study, there was no association between BP and DFU or amputations. Previous studies reported conflicting results for the association between BP and DFU.^8^ One study found a higher proportion of individuals with hypertension among those with DFU,^1^ another study reported a higher risk for amputation in individuals with >10 years of hypertension from the diagnoses of DFU,^32^ and other studies found no significant association between DFU or amputations and hypertension.^11^, ^12^ Nevertheless, the proportion of people with DFU who were treated with antihypertensive medication was markedly increased. This could be due to preceding hypertension that might have driven the development of foot ulceration in these individuals who now are treated according to guidelines^33^ and therefore differences in the proportion of hypertension are difficult to detect.

The findings on smoking and alcohol consumption were ambiguous as well. At least in T2D and for smoking in males with T1D, these habits were related to the occurrence of DFU, but no clear risk enhancement for amputations could be detected after adjustment. This is surprising, at least for smoking, as this is one of the few widely confirmed risk factors for amputations,^8^, ^11^, ^34^ while there is less evidence concerning alcohol consumption.^12^, ^35^ Our results might have been mitigated due to the self‐reported manner in which these behaviors are documented. Another possibility might be that once having developed a DFU, quitting smoking or drinking alcohol does no more to protect from amputations.

Insulin pump use was associated with less DFU in T1D, and this association remained with adjustment for BMI and HbA1c. We could not find previous studies reporting a relation between multiple daily injections or insulin pump use and DFU, but a Swedish cohort study found that HbA1c variability was associated with cardiovascular diseases^36^ and pump use might reduce this variability. Another study investigated wound healing of DFU in T2D, which significantly improved from insulin pump use compared to multiple daily injections.^37^ In T2D, insulin was more often prescribed in adults with DFU, which confirms findings from other studies and might relate to a higher disease burden and more requirement for nursing in these individuals.^8^, ^38^, ^39^ This is supported by a Chinese study that found higher risk for DFU in individuals with severe insulin‐deficient diabetes, a group of mostly insulin‐dependent individuals with T2D (88%) the researchers identified via cluster analyses.^40^ SGLT2i are under suspicion to enhance the risk for lower limb amputations,^41^, ^42^, ^43^ but there are also studies questioning this.^44^, ^45^ A recent meta‐analysis concluded that GLP‐1RA might be advantageous to the other oral glucose lowering agent; dipeptidyl peptidase‐4 inhibitors (DPP4i), but SGLT2i have no positive or adverse association to amputations.^46^ This would be in line with our results revealing that treatment with metformin or GLP‐1RA in the year preceding the first DFU documentation was associated with lower hazard ratios for amputations compared to insulin therapy and to other oral hypoglycemic medication even with adjustment for HbA1c categories. Further, the higher crude HRs for amputation among those on SGLT2i were no longer significant after adjustment for age, sex, diabetes duration, and treatment year. This might indicate that the patient selection of SGLT2i treatment might be mainly responsible for this effect rather than the pharmacologic treatment itself.

The strength of the present study is that a high number of individuals with T1D and T2D could be observed over a long treatment period enabling us to affirm or question existing assumptions. A prospective, standardized electronic documentation was used. Therefore, possible associations to diabetic foot ulcers and lower limb amputations could be evaluated with real‐world data from 255 institutions including over 60 000 individuals in the matched cohort. Limitations include the lack of other classification systems for DFU severity that consider infection status as well, such as the University of Texas wound classification scheme (available in only ≈1% of individuals) or the SINBAD (Site, Ischemia, Bacterial infection, Area and Depth) classification which is the currently recommended classification system by the International Working Group on the Diabetic Foot.^47^ Nevertheless, Wagner grades are still recommended by the German Diabetes Association^21^ and therefore this is standard for centers participating in the DPV registry and used for this analysis. The self‐reported manner of the documentation of alcohol consumption and smoking behavior is also a potential bias. Information on specific nutritional habits of the participants were not available, unfortunately. Further, we did not include socioeconomic indicators as these would have been available only for Germany and on a regional level, as individual socioeconomic status is not available in the registry. Additionally, we did not have standardized information on the exact structure of each center regarding a multidisciplinary team. Results on medication and therapy should be interpreted with caution as many different combinations of diabetes and nondiabetes medications as well as therapy changes are likely in different individuals. Additionally, no causal conclusions can be drawn from our observational study. Due to the distribution of individuals with DFU over the years, we had to match the control group by calendar year to avoid bias because of medications that were not yet or no longer available for prescription. Therefore, the matched study population includes many individuals treated several years back and does not perfectly describe the current proportions of medications and therapies.

## 5 CONCLUSION

Risk assessment for DFU is dependent on similar characteristics in adults with T1D and T2D with male sex, taller body height, and comorbidities such as neuropathy, nephropathy, PAD, and retinopathy identifying individuals with higher risk for DFU. Risk factors amenable to intervention differ partly between diabetes types. Although improvement of glycemic control and lipid levels seems to be important in T1D, smoking and drinking behavior as well as type of diabetes medication should be considered in T2D.

## AUTHOR CONTRIBUTIONS

Alexander J. Eckert analyzed the data and wrote the first draft of the paper. Reinhard W. Holl conceived and coordinated the study and reviewed the article critically. The authors Stefan Zimny, Marcus Altmeier, Ana Dugice, Anton Gillessen, Latife Bozkurt, Gabriele Götz, Wolfram Karges, Frank J. Wosch, and Stephan Kress participated in the DPV initiative by providing data and reviewed the article critically.

## FUNDING INFORMATION

This study was supported through the German Federal Ministry for Education and Research within the German Centre for Diabetes Research (82DZD14E03). Further financial support was received by the German Robert Koch Institute and by the German Diabetes Association. Sponsors were not involved in data acquisition or analysis.

## DISCLOSURE

None.

## Supporting information


**Data S1.** Supplementary list 1. List of DPV (Diabetes‐Patienten‐Verlaufsdokumentation) centers contributing to this analysis.

**Figure S1.** Selection criteria and groups for analyses. * Individuals with diabetic foot ulcers (DFU) before the year 2000 or the age of 18 years and individuals with a documentation of DFU to an unknown time were excluded from the analysis.

**Figure S2.** Hazard ratios for the risk of amputation in individuals with diabetic foot ulcers with specific medication for type 2 diabetes (T2D). All individuals were followed for at least 100 days and initial amputations within the first 100 days after the first documentation of diabetic foot ulcer were excluded. Hazard ratios were calculated in a complete model including all shown parameters and adjusted for age, sex, diabetes duration, and calendar year.

**Table S1.** Mean differences and standardized differences of matching variables and propensity score before and after greedy matching.

## References

[1] Zhang P , Lu J , Jing Y , Tang S , Zhu D , Bi Y . Global epidemiology of diabetic foot ulceration: a systematic review and meta‐analysis. Ann Med. 2017;49(2):106‐116. 27585063 10.1080/07853890.2016.1231932

[2] Bandyk DF . The diabetic foot: pathophysiology, evaluation, and treatment. Semin Vasc Surg. 2018;31(2–4):43‐48. 30876640 10.1053/j.semvascsurg.2019.02.001

[3] Boulton AJM , Vileikyte L , Ragnarson‐Tennvall G , Apelqvist J . The global burden of diabetic foot disease. Lancet. 2005;366(9498):1719‐1724. 16291066 10.1016/S0140-6736(05)67698-2

[4] Van GH , Amouyal C , Bourron O , et al. Diabetic foot ulcer management in a multidisciplinary foot centre: one‐year healing, amputation and mortality rate. J Wound Care. 2021;30(Sup 6):S34‐S41. 34120465 10.12968/jowc.2021.30.Sup6.S34

[5] Armstrong DG , Swerdlow MA , Armstrong AA , Conte MS , Padula WV , Bus SA . Five year mortality and direct costs of care for people with diabetic foot complications are comparable to cancer. J Foot Ankle Res. 2020;13(1):16. 32209136 10.1186/s13047-020-00383-2 PMC7092527

[6] Sen P , Demirdal T , Emir B . Meta‐analysis of risk factors for amputation in diabetic foot infections. Diabetes Metab Res Rev. 2019;35(7):e3165. 30953392 10.1002/dmrr.3165

[7] Lu Q , Wang J , Wei X , Wang G , Xu Y . Risk factors for major amputation in diabetic foot ulcer patients. Diabetes Metab Syndr Obes. 2021;14:2019‐2027. 33976562 10.2147/DMSO.S307815 PMC8106455

[8] Rossboth S , Lechleitner M , Oberaigner W . Risk factors for diabetic foot complications in type 2 diabetes‐a systematic review. Endocrinol Diabetes Metab. 2021;4(1):e00175. 33532615 10.1002/edm2.175 PMC7831214

[9] Li X , Xiao T , Wang Y , et al. Incidence, risk factors for amputation among patients with diabetic foot ulcer in a Chinese tertiary hospital. Diabetes Res Clin Pract. 2011;93(1):26‐30. 21466901 10.1016/j.diabres.2011.03.014

[10] Boyko EJ , Zelnick LR , Braffett BH , et al. Risk of foot ulcer and lower‐extremity amputation among participants in the diabetes control and complications trial/epidemiology of diabetes interventions and complications study. Diabetes Care. 2022;45(2):357‐364. 35007329 10.2337/dc21-1816 PMC8914413

[11] Lin C , Liu J , Sun H . Risk factors for lower extremity amputation in patients with diabetic foot ulcers: a meta‐analysis. PLoS One. 2020;15(9):e0239236. 32936828 10.1371/journal.pone.0239236 PMC7494323

[12] Wang L , Li Q , Chen X , Wang Z . Clinical characteristics and risk factors of lower extremity amputation in patients with diabetic foot. Pak J Med Sci. 2022;38(8):2253‐2258. 36415262 10.12669/pjms.38.8.5635 PMC9676613

[13] Austin PC . An introduction to propensity score methods for reducing the effects of confounding in observational studies. Multivariate Behav Res. 2011;46(3):399‐424. 21818162 10.1080/00273171.2011.568786 PMC3144483

[14] Schleicher E , Gerdes C , Petersmann A , et al. Definition, classification and diagnosis of diabetes mellitus. Exp Clin Endocrinol Diabetes. 2022;130:S1‐S8. 35451038 10.1055/a-1624-2897

[15] Rosenbauer J , Dost A , Karges B , et al. Improved metabolic control in children and adolescents with type 1 diabetes: a trend analysis using prospective multicenter data from Germany and Austria. Diabetes Care. 2012;35(1):80‐86. 22074726 10.2337/dc11-0993 PMC3241332

[16] Eckert AJ , Linke S , Schwab K‐O , et al. Changes in cardiovascular risk factors among children and young adults with type 1 diabetes during the COVID‐19 pandemic compared to previous years‐results from the German DPV registry. J Diabetes. 2023;15(1):15‐26. 36621521 10.1111/1753-0407.13340 PMC9870744

[17] Alberti KG , Zimmet P , Shaw J . Metabolic syndrome—a new world‐wide definition. A consensus statement from the international diabetes federation. Diabet Med. 2006;23(5):469‐480. 16681555 10.1111/j.1464-5491.2006.01858.x

[18] Batra A , Müller CA , Mann K , Heinz A . Alcohol dependence and harmful use of alcohol. Dtsch Arztebl Int. 2016;113(17):301‐310. 27173413 10.3238/arztebl.2016.0301 PMC4873678

[19] Hammes H‐P , Welp R , Kempe H‐P , Wagner C , Siegel E , Holl RW . Risk factors for retinopathy and DME in type 2 diabetes‐results from the German/Austrian DPV database. PloS One. 2015;10(7):e0132492. 26177037 10.1371/journal.pone.0132492 PMC4503301

[20] Valente MAE , Hillege HL , Navis G , et al. The chronic kidney disease epidemiology collaboration equation outperforms the modification of diet in renal disease equation for estimating glomerular filtration rate in chronic systolic heart failure. Eur J Heart Fail. 2014;16(1):86‐94. 23901055 10.1093/eurjhf/hft128

[21] Morbach S , Eckhard M , Lobmann R , et al. Diabetisches Fußsyndrom. Diabetologie Und Stoffwechsel. 2022;17:S365‐S375.

[22] Reitzle L , Schmidt C , Du Y , et al. Einschätzungen zur Prävalenz mikrovaskulärer Folgeerkrankungen bei Diabetes mellitus in Deutschland. Analyse von Versichertendaten aller gesetzlichen Krankenkassen für die Jahre 2012 und 2013. Bundesgesundheitsblatt Gesundheitsforschung Gesundheitsschutz. 2020;63(10):1219‐1230. 32876717 10.1007/s00103-020-03211-x

[23] Bohn B , Grünerbel A , Altmeier M , et al. Diabetic foot syndrome in patients with diabetes. A multicenter German/Austrian DPV analysis on 33 870 patients. Diabetes Metab Res Rev. 2018;34(6):e3020. 29726089 10.1002/dmrr.3020

[24] Lane KL , Abusamaan MS , Voss BF , et al. Glycemic control and diabetic foot ulcer outcomes: a systematic review and meta‐analysis of observational studies. J Diabetes Complications. 2020;34(10):107638. 32527671 10.1016/j.jdiacomp.2020.107638 PMC7721205

[25] Musuuza J , Sutherland BL , Kurter S , Balasubramanian P , Bartels CM , Brennan MB . A systematic review of multidisciplinary teams to reduce major amputations for patients with diabetic foot ulcers. J Vasc Surg. 2020;71(4):1433‐1446.e3. 31676181 10.1016/j.jvs.2019.08.244 PMC7096268

[26] Stettler C , Allemann S , Jüni P , et al. Glycemic control and macrovascular disease in types 1 and 2 diabetes mellitus: meta‐analysis of randomized trials. Am Heart J. 2006;152(1):27‐38. 16824829 10.1016/j.ahj.2005.09.015

[27] Eckert AJ , Plaumann M , Pehlke S , et al. Idiopathic frozen shoulder in individuals with diabetes: association with metabolic control, obesity, antidiabetic treatment and demographic characteristics in adults with type 1 or 2 diabetes from the DPV registry. Exp Clin Endocrinol Diabetes. 2022;130(7):468‐474. 34425597 10.1055/a-1543-8559

[28] Merger SR , Kerner W , Stadler M , et al. Prevalence and comorbidities of double diabetes. Diabetes Res Clin Pract. 2016;119:48‐56. 27449710 10.1016/j.diabres.2016.06.003

[29] Pastore D , Deja‐Simoni A , De Stefano A , et al. Risk factors for diabetic foot ulcers: an Albanian retrospective study of inpatients with type 2 diabetes. Eur Rev Med Pharmacol Sci. 2022;26(2):558‐572. 35113432 10.26355/eurrev_202201_27883

[30] Tang WH , Zhao YN , Cheng ZX , Xu JX , Zhang Y , Liu XM . Risk factors for diabetic foot ulcers: a systematic review and meta‐analysis. Vascular. 2023. doi:10.1177/17085381231154805 36740805

[31] Ulloque‐Badaracco JR , Mosquera‐Rojas MD , Hernandez‐Bustamante EA , et al. Association between lipid profile and apolipoproteins with risk of diabetic foot ulcer: a systematic review and meta‐analysis. Int J Clin Pract. 2022;2022:5450173. 36016824 10.1155/2022/5450173 PMC9385316

[32] Rossboth S , Rossboth B , Schoenherr H , Ciardi C , Lechleitner M , Oberaigner W . Diabetic foot complications‐lessons learned from real‐world data derived from a specialized Austrian hospital. Wien Klin Wochenschr. 2022;134(1–2):7‐17. 33938984 10.1007/s00508-021-01864-5

[33] de Boer IH , Bangalore S , Benetos A , et al. Diabetes and hypertension: a position statement by the American Diabetes Association. Diabetes Care. 2017;40(9):1273‐1284. 28830958 10.2337/dci17-0026

[34] Naemi R , Chockalingam N , Lutale JK , Abbas ZG . Predicting the risk of future diabetic foot ulcer occurrence: a prospective cohort study of patients with diabetes in Tanzania. BMJ Open Diabetes Res Care. 2020;8(1):e001122. 10.1136/bmjdrc-2019-001122 PMC7228475 32371531

[35] Lee YJ , Han K‐D , Kim JH . Association among current smoking, alcohol consumption, regular exercise, and lower extremity amputation in patients with diabetic foot: Nationwide population‐based study. Endocrinol Metab (Seoul). 2022;37(5):770‐780. 36222086 10.3803/EnM.2022.1519 PMC9633221

[36] Ceriello A , Lucisano G , Prattichizzo F , et al. HbA1c variability predicts cardiovascular complications in type 2 diabetes regardless of being at glycemic target. Cardiovasc Diabetol. 2022;21(1):13. 35073913 10.1186/s12933-022-01445-4 PMC8788128

[37] Fan HJ , Yu JH , Cui GM , Zhang WY , Yang X , Dong QJ . Insulin pump for the treatment of diabetes in combination with ulcerative foot infections. J Biol Regul Homeost Agents. 2016;30(2):465‐470. 27358133

[38] Banik PC , Barua L , Moniruzzaman M , Mondal R , Zaman F , Ali L . Risk of diabetic foot ulcer and its associated factors among Bangladeshi subjects: a multicentric cross‐sectional study. BMJ Open. 2020;10(2):e034058. 10.1136/bmjopen-2019-034058 PMC7050319 32114471

[39] Yazdanpanah L , Shahbazian H , Nazari I , et al. Incidence and risk factors of diabetic foot ulcer: a population‐based diabetic foot cohort (ADFC study)‐two‐year follow‐up study. Int J Endocrinol. 2018;2018:7631659. 29736169 10.1155/2018/7631659 PMC5875034

[40] Xiong X‐F , Yang Y , Wei L , Xiao Y , Li L , Sun L . Identification of two novel subgroups in patients with diabetes mellitus and their association with clinical outcomes: a two‐step cluster analysis. J Diabetes Investig. 2021;12(8):1346‐1358. 10.1111/jdi.13494 PMC8354513 33411406

[41] Chang H‐Y , Singh S , Mansour O , Baksh S , Alexander GC . Association between sodium‐glucose cotransporter 2 inhibitors and lower extremity amputation among patients with type 2 diabetes. JAMA Intern Med. 2018;178(9):1190‐1198. 30105373 10.1001/jamainternmed.2018.3034 PMC6142968

[42] Li D , Yang JY , Wang T , Shen S , Tang H . Risks of diabetic foot syndrome and amputation associated with sodium glucose co‐transporter 2 inhibitors: a meta‐analysis of randomized controlled trials. Diabetes Metab. 2018;44(5):410‐414. 29506779 10.1016/j.diabet.2018.02.001

[43] Matthews DR , Li Q , Perkovic V , et al. Effects of canagliflozin on amputation risk in type 2 diabetes: the CANVAS program. Diabetologia. 2019;62(6):926‐938. 30868176 10.1007/s00125-019-4839-8 PMC6509073

[44] Du Y , Bai L , Fan B , et al. Effect of SGLT2 inhibitors versus DPP4 inhibitors or GLP‐1 agonists on diabetic foot‐related extremity amputation in patients with T2DM: a meta‐analysis. Prim Care Diabetes. 2022;16(1):156‐161. 34930687 10.1016/j.pcd.2021.12.007

[45] Heyward J , Mansour O , Olson L , Singh S , Alexander GC . Association between sodium‐glucose cotransporter 2 (SGLT2) inhibitors and lower extremity amputation: a systematic review and meta‐analysis. PLoS One. 2020;15(6):e0234065. 32502190 10.1371/journal.pone.0234065 PMC7274434

[46] Scheen AJ . Lower limb amputations: protection with GLP‐1 receptor agonists rather than increased risk with SGLT2 inhibitors? Diabetes Metab. 2022;48(2):101325. 35121148 10.1016/j.diabet.2022.101325

[47] Monteiro‐Soares M , Hamilton EJ , Russel DA , et al. Guidelines on the classification of foot ulcers in people with diabetes IWGDF 2023 update. Diabetes Metab Res Rev. 2023;2023:71‐101. 10.1002/dmrr.3648 37179483

