# Supplementary figures and images for: Factors associated with diabetic foot ulcers and lower limb amputations in type 1 and type 2 diabetes supported by real‐world data from the German/Austrian DPV registry
Source: J Diabetes. 2024 Feb 25;16(2):e13531. doi: 10.1111/1753-0407.13531 (PMC10894714; doi:10.1111/1753-0407.13531)

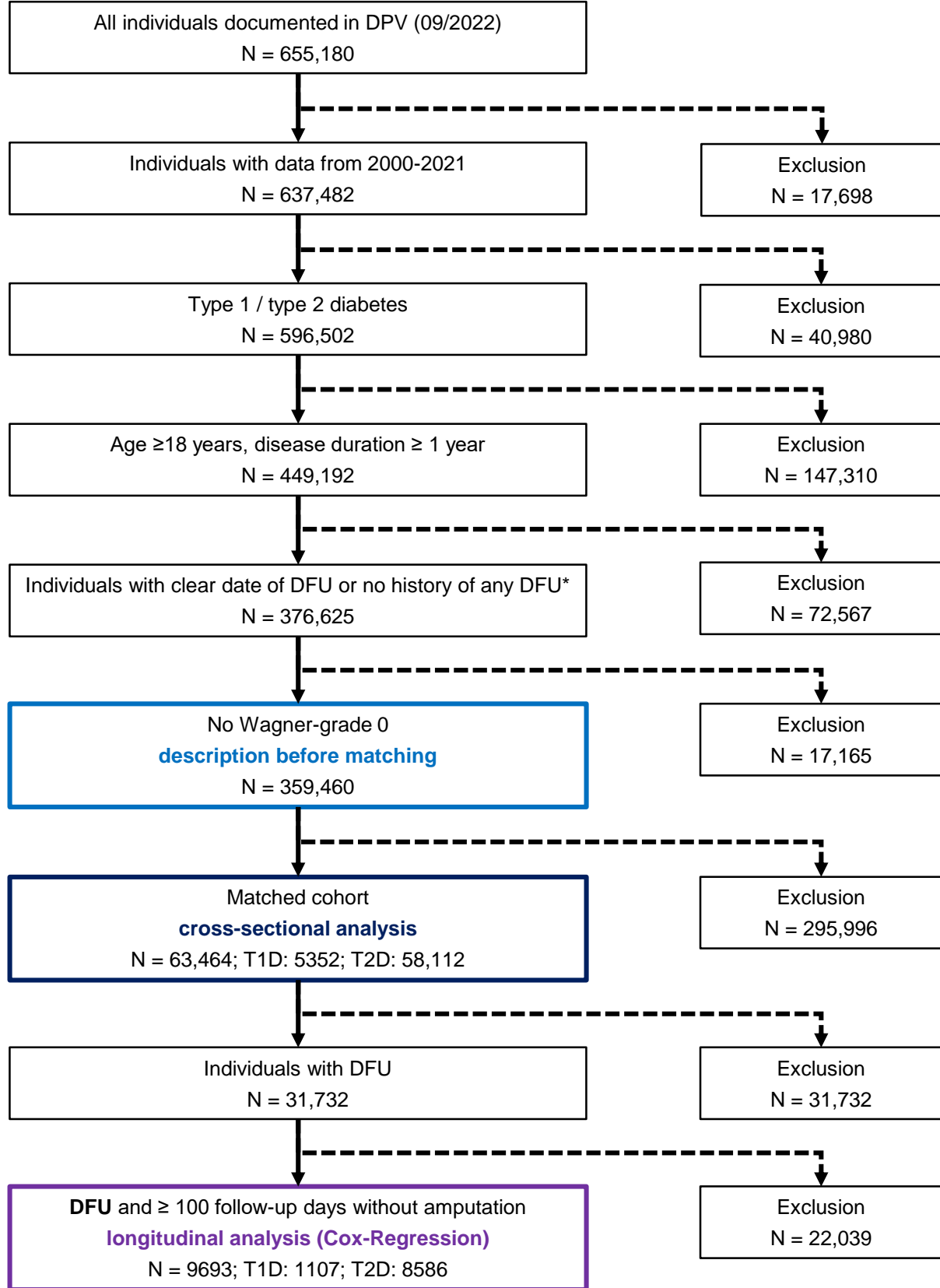

Supplement: Supplementary file 2 — Figure S1. Selection criteria and groups for analyses. * Individuals with diabetic foot ulcers (DFU) before the year 2000 or the age of 18 years and individuals with a documentation of DFU to an unknown time were excluded from the analysis. [file JDB-16-e13531-s004.pdf]

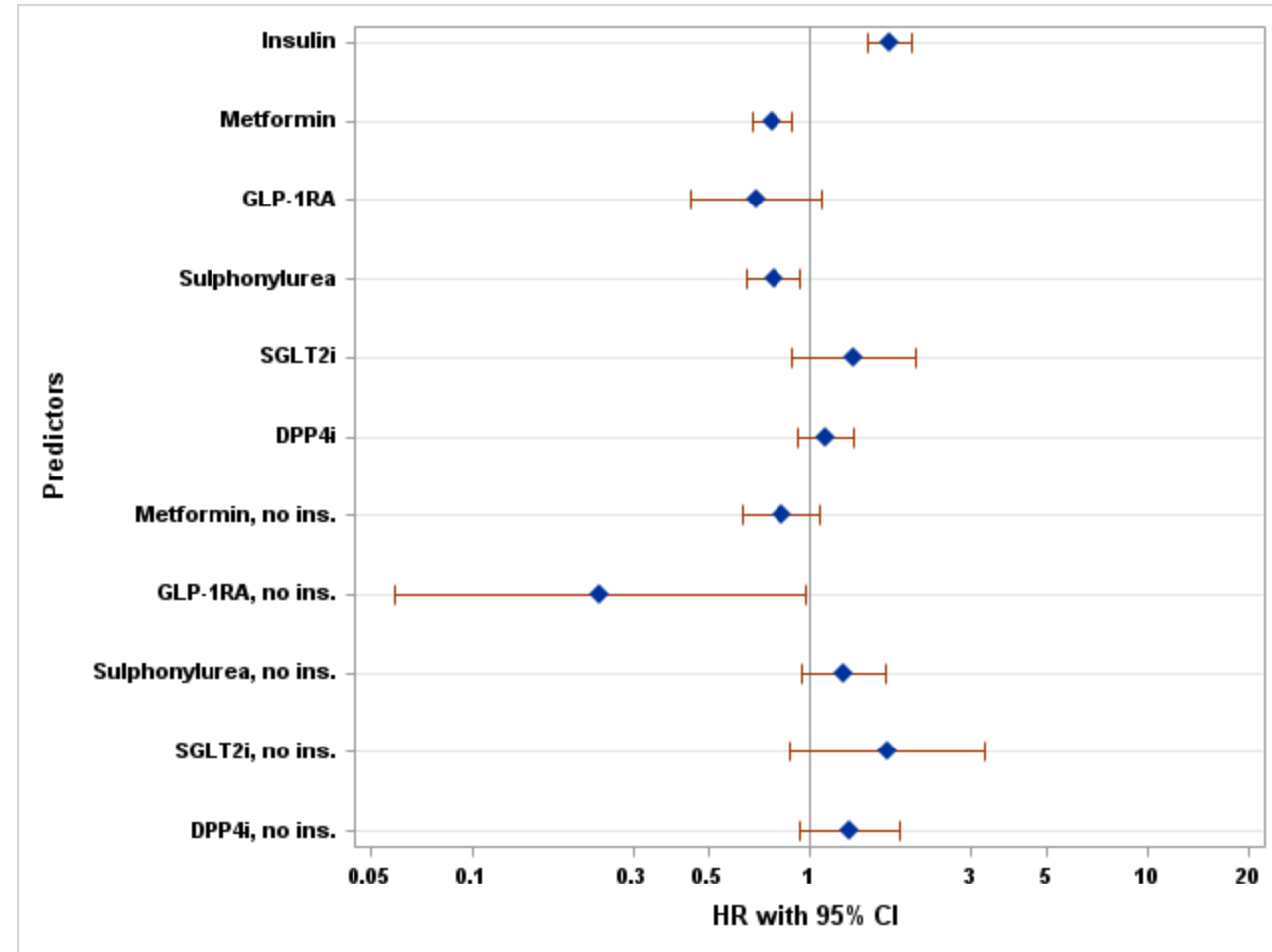

Supplement: Supplementary file 3 — Figure S2. Hazard ratios for the risk of amputation in individuals with diabetic foot ulcers with specific medication for type 2 diabetes (T2D). All individuals were followed for at least 100 days and initial amputations within the first 100 days after the first documentation of diabetic foot ulcer were excluded. Hazard ratios were calculated in a complete model including all shown parameters and adjusted for age, sex, diabetes duration, and calendar year. [file JDB-16-e13531-s002.pdf]
