# Supplementary material for: Factors associated with diabetic foot ulcers and lower limb amputations in type 1 and type 2 diabetes supported by real‐world data from the German/Austrian DPV registry
Source: J Diabetes. 2024 Feb 25;16(2):e13531. doi: 10.1111/1753-0407.13531 (PMC10894714; doi:10.1111/1753-0407.13531)
Supplement: Supplementary file 4 — Table S1. Mean differences and standardized differences of matching variables and propensity score before and after greedy matching. [file JDB-16-e13531-s003.docx]

***Supplementary Table S1.****Mean differences and standardized differences of matching variables and propensity score before and after greedy matching.*

| **Matching Variable** | **Mean difference** | | **Standardized difference** | |
| --- | --- | --- | --- | --- |
| **Type 1 diabetes** | **before matching** | **after matching** | **before matching** | **after matching** |
| **Sex (proportion male)** | -0.09065 | 0 | -0.18382 | 0 |
| **Age [years]** | 20.90229 | -0.15343 | 1.21434 | -0.00891 |
| **Diabetes duration [years]** | 10.65006 | 0.14082 | 0.76604 | 0.01013 |
| **Year** | -3.41803 | 0 | -0.59818 | 0 |
| **Propensity Score** | 2.36428 | -0.00296 | 1.4902 | -0.00187 |
| **Type 2 diabetes** | **before matching** | **after matching** | **before matching** | **after matching** |
| **Sex (proportion male)** | -0.1316 | 0 | -0.26871 | 0 |
| **Age [years]** | 1.11964 | -0.19984 | 0.09438 | -0.01685 |
| **Diabetes duration [years]** | 2.73229 | 0.09272 | 0.29712 | 0.01008 |
| **Year** | -1.49318 | 0 | -0.28079 | 0 |
| **Propensity Score** | 0.32276 | -0.00004 | 0.57094 | -0.00008 |
